# Nanoengineering Carboxysome Shells for Protein Cages with Programmable Cargo Targeting

**DOI:** 10.1021/acsnano.3c11559

**Published:** 2024-02-07

**Authors:** Tianpei Li, Ping Chang, Weixian Chen, Zhaoyang Shi, Chunling Xue, Gregory F. Dykes, Fang Huang, Qiang Wang, Lu-Ning Liu

**Affiliations:** † State Key Laboratory of Crop Stress Adaptation and Improvement, School of Life Sciences, Henan University, Kaifeng 475004, China; ‡ Institute of Systems, Molecular and Integrative Biology, University of Liverpool, Liverpool L69 7ZB, United Kingdom; § MOE Key Laboratory of Evolution and Marine Biodiversity, Frontiers Science Center for Deep Ocean Multispheres and Earth System & College of Marine Life Sciences, Ocean University of China, Qingdao 266003, China

**Keywords:** bacterial microcompartment, carboxysome, protein shell, cargo loading, nanocage, self-assembly, synthetic biology

## Abstract

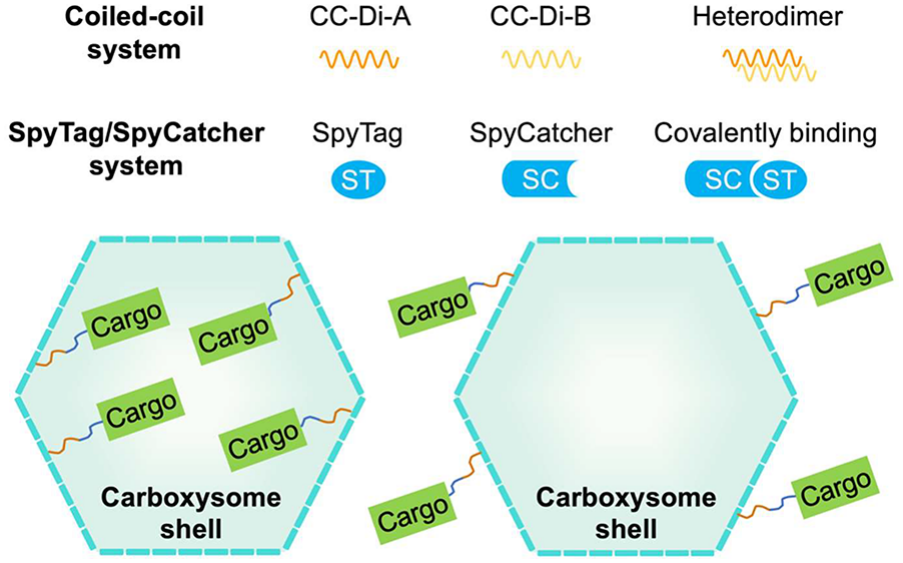

Protein nanocages have emerged as promising candidates for enzyme immobilization and cargo delivery in biotechnology and nanotechnology. Carboxysomes are natural proteinaceous organelles in cyanobacteria and proteobacteria and have exhibited great potential in creating versatile nanocages for a wide range of applications given their intrinsic characteristics of self-assembly, cargo encapsulation, permeability, and modularity. However, how to program intact carboxysome shells with specific docking sites for tunable and efficient cargo loading is a key question in the rational design and engineering of carboxysome-based nanostructures. Here, we generate a range of synthetically engineered nanocages with site-directed cargo loading based on an α-carboxysome shell in conjunction with SpyTag/SpyCatcher and Coiled-coil protein coupling systems. The systematic analysis demonstrates that the cargo-docking sites and capacities of the carboxysome shell-based protein nanocages could be precisely modulated by selecting specific anchoring systems and shell protein domains. Our study provides insights into the encapsulation principles of the α-carboxysome and establishes a solid foundation for the bioengineering and manipulation of nanostructures capable of capturing cargos and molecules with exceptional efficiency and programmability, thereby enabling applications in catalysis, delivery, and medicine.

Subcellular compartmentalization provides the framework for spatially sequestering multiple concurrent metabolic processes within cells and facilitating their performance and functional coordination. The well-known paradigms of cellular compartmentalization encompass membrane-bound organelles such as mitochondria, lysosomes, and peroxisomes in eukaryotic cells. Emerging evidence has now demonstrated that prokaryotes have evolved proteinaceous organelle-like compartments, known as bacterial microcompartments (BMCs), to sequester incompatible biochemical pathways involving toxic or volatile intermediates and optimize metabolic reactions.^1−3^ The BMC comprises a core of cargo enzymes encapsulated by a polyhedral shell. The shell is constructed by a series of homologous shell proteins that are mainly in the forms of hexamers, pentamers, and trimers through self-assembly and contain a central pore, providing a physical barrier facilitating cargo encapsulation while offering selective permeability.^4,5^ Due to their inherent self-assembling and architectural properties, BMCs possess exceptional potential for designing and generating artificial metabolic nanoreactors and scaffolding/delivery systems by orchestrating enzymes and molecules within the protein organelles.^6−10^

Carboxysomes (CBs) are CO_2_-fixing BMCs found in all cyanobacteria and some chemoautotrophs.^11^ The CB encapsulates the CO_2_-fixing enzyme, ribulose-1,5-bisphosphate carboxylase/oxygenase (Rubisco), and carbonic anhydrase (CA) within the polyhedral protein shell.^12−17^ The semipermeable shell permits the entry of HCO_3_^–^ while restraining the passage of CO_2_, which is subsequently converted from HCO_3_^–^ by the interior CA, resulting in high levels of CO_2_ within the shell for facilitating Rubisco carboxylation and diminishing photorespiration.^18−21^ CBs can be categorized into two lineages: α-CBs and β-CBs, which differ in the phylogenetic subclass of Rubisco enclosed proteins and their protein composition. Moreover, unlike β-CBs that undertake “Cargo first” assembly pathway,^22,23^ the self-assembly of α-CBs follows a “Shell first” or “Concomitant shell–core assembly” mode.^24−26^ CsoS2 has been demonstrated to play an essential role in the assembly of the α-CB by binding with Rubisco using its N-terminus^27^ and forming strong interactions with the shell inner surface through its C-terminus (CsoS2-C).^28^ Advanced knowledge of α-CB formation has facilitated rational engineering and manipulation of α-CBs and empty α-CB shells that have the potential to encase heterologous cargos. Previous studies have demonstrated the possibility of synthetically engineering CBs and empty α-CB shells in *E. coli*(9,29,30) as well as encapsulating non-native cargos into the α-CB shells to construct nanobioreactors for specific functions.^9,31^

Despite their great potential in diverse biotechnological applications, a challenge in engineering BMC-based organelles or scaffolding systems is the establishment of efficient, site-directed cargo encapsulation. To address this issue, several cargo-loading strategies have been explored for recruiting cargo proteins into BMCs: (i) fusion of endogenous encapsulation peptides (EPs) to target cargos;^6,9,31−35^ (ii) fusion of foreign proteins to the termini of major shell proteins;^36^ and (iii) integration of anchoring peptides (AP), such as the SpyTag/SpyCatcher (ST/SC) or Coiled-coil systems, into major shell proteins and cargos for specific binding.^37−41^ However, a systematic analysis of the cargo-loading strategies to discern efficient and adjustable cargo-docking modules has not been established, which limits further engineering and development of BMC-based caging systems.

Here, we performed *de novo* design to generate a series of protein nanocages with site-directed cargo recruitment based on an α-CB shell and protein–protein coupling systems. We then conducted a systematic assessment of the cargo-loading capacities of the engineered nanocages mediated by various cargo-directing strategies. Our findings shed insights into the encapsulation mechanisms of α-CB shells and provide a solid groundwork for the strategic formulation and crafting of α-CB- or BMC-derived nanocages for optimal and tunable cargo capture and encapsulation to facilitate diverse biotechnological and biomedical applications.

## Results and Discussion

### Generation of α-CB Shells Incorporated with Synthetic Anchoring Peptides

Among the reported cargo-loading strategies, fusing proteins directly to the termini of major shell proteins may interfere with the self-assembly of BMC shells.^36^ Endogenous EPs tend to form aggregates owing to their disordered structures^42^ or may offer a relatively low cargo-loading capability due to their finite binding sites on the inner surface of the protein shell and limited interactions with shell proteins. CsoS2 plays a crucial role in the formation of the α-CB shell by interacting with shell proteins on the inner surface of the shell through CsoS2-C.^28^ Based on this encapsulation mechanism, we have shown that CsoS2-C can serve as an EP to recruit non-native cargo proteins into the recombinant α-CB shells derived from the chemoautotrophic bacterium *Halothiobacillus neapolitanus*.^9,21,31^ However, there are only 192 copies of CsoS2B (the full-length CsoS2 that contains CsoS2-C) in contrast to 986 copies of shell hexamers and pentamers in the native α-CB from *H. neapolitanus*,^17^ suggesting the limited inherent capacity of CsoS2-C for recruiting cargos into the α-CB shell. Moreover, the CsoS2-C EP peptides that are fused with foreign proteins for cargo recruitment would inevitably compete with the native CsoS2 polypeptides that drive the formation of the α-CB shell for the limited docking site on the shell inner surface.

**Figure 1:**
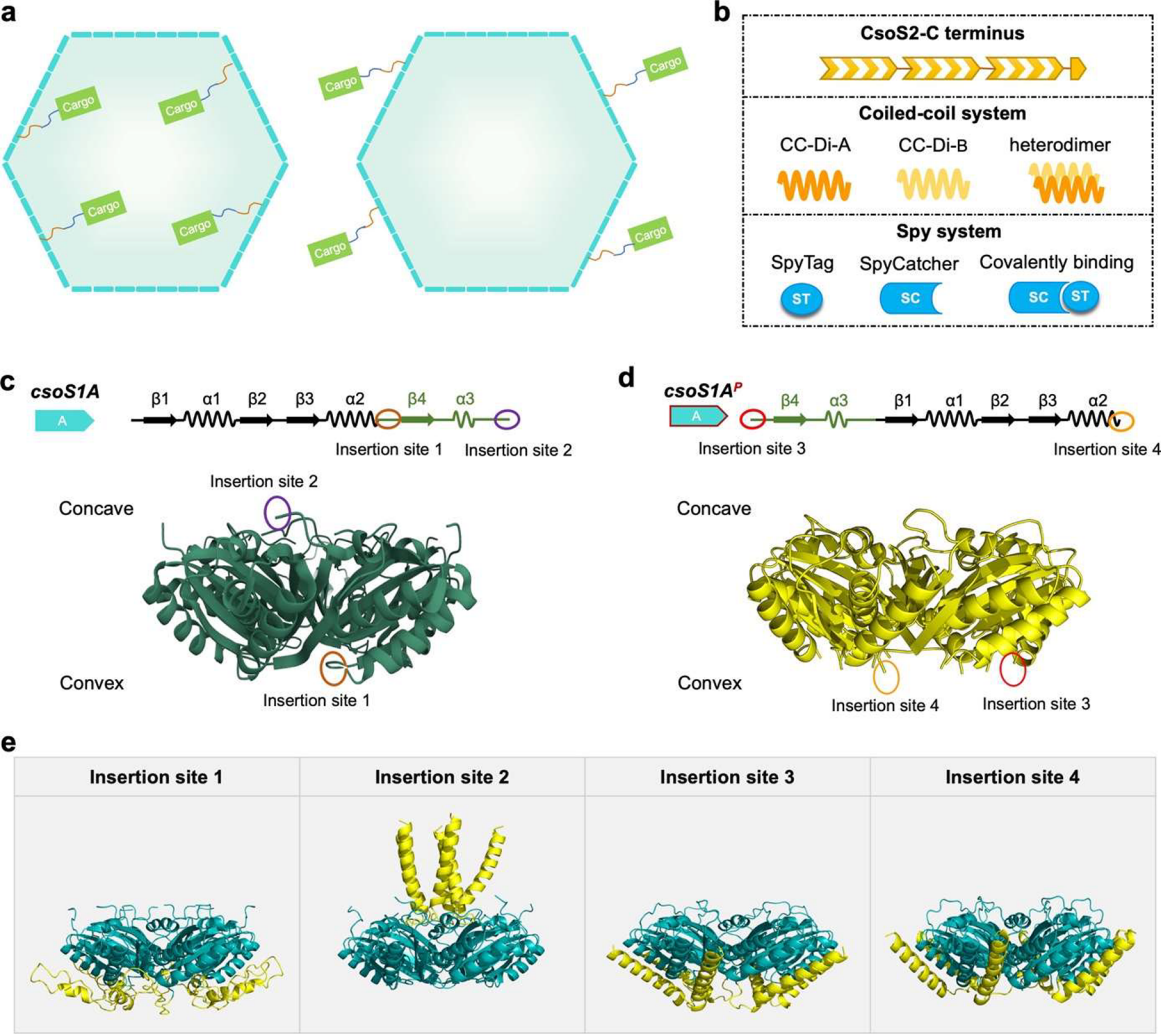
Strategies for constructing tunable carboxysome shell-based cargo-loading platforms. (a) Cartoon models of cargo-loaded CB shells with cargo encapsulated inside the shell or attached on the outer surface of the shell. (b) Three candidate peptides or peptide pairs could be employed for cargo-loading. CsoS2-C serves as an encapsulation peptide to direct foreign proteins inside the shell. The Coiled-coil system is composed of CC^A^ and CC^B^ motifs, which can form a heterodimer through electrostatic and hydrophobic interactions at the peptide interface. The SpyTag/SpyCatcher (ST/SC) system consists of ST and SC, which can covalently bind with each other. (c) Primary structure of wild-type CsoS1A and side view of the model of the CsoS1A hexamer (PDB code 2G13). Purple and orange circles indicate the concave side-facing C-terminus (potential AP insertion site 2) and convex side-facing region between the second α-helix and the fourth β-sheet (insertion site 1), respectively. (d) Primary structure of circularly permuted CsoS1A (CsoS1A^P^) and AlphaFold-predicted structure of CsoS1A^P^ hexamer in which the N-terminus (insertion site 3) and C-terminus (insertion site 4) indicated by red and yellow circles are located at the convex side. CsoS1A^P^ was generated by relocating the C-terminal region (green) of native CsoS1A and was transplanted to its N-terminus. (e) AlphaFold-predicted structures of four types of CsoS1A/CsoS1A^P^ hexamers with CC^A^ fused at each insertion site as illustrated in (c) and (d). The CC^A^ peptide is colored yellow.

In contrast, insertion of a synthetic AP into the shell proteins, along with producing cargo enzymes of interest fused with the cognate interacting counterpart of AP, could ensure the physical proximity and site-specific encapsulation of cargos with controlled stoichiometry (Figure 1a,b). Two sets of protein–protein coupling systems, the Coiled-coil system and ST/SC system, have been utilized for targeting exogenous cargos to recombinant BMCs.^37,39−41,43−46^ The Coiled-coil system is made up of two orthogonal peptides, CC-Di-A (2.3 kDa, hereafter denoted as CC^A^) and CC-Di-B (2.3 kDa, hereafter denoted as CC^B^), which can form a highly stable heterodimer through electrostatic and hydrophobic interactions.^47^ This system has been exploited to incorporate cargos inside 1,2-propanediol-utilization (Pdu) BMCs.^37,41^ The ST/SC system takes advantage of the SpyTag (ST, 1.5 kDa) and the cognate SpyCatcher (SC, 9.1 kDa) peptides that can form covalent interactions, thereby mediating the colocalization of proteins linked with ST and SC, respectively.^43−45^

To establish site-directed cargo recruitment on the recombinant hollow α-CB shell (Figure 1a), we employed the AP-based cargo-loading strategies using the heterodimeric CC^A^/CC^B^ peptides^48^ and the ST/SC peptide pair^49^ (Figure 1b). The main shell protein, CsoS1A (PDB code 2G13), was selected as the anchoring target for conjugating with AP (Figure 1c), as CsoS1A/C are the major shell proteins, accounting for more than 60% of total shell proteins in the native and recombinant α-CBs.^17^ The N- and C-termini of wild-type (WT) CsoS1A are located on the concave side of the hexamer and face the cellular cytoplasm^50−52^ (Figure 1c). Thus, insertion site 2 is expected to target cargo proteins on the outer surface of the α-CB shell. In contrast, the region between α-helix 2 (α2) and β-sheet 4 (β4) of CsoS1A faces the luminal side without any contact with neighboring shell proteins (Figure 1c).^50^ Therefore, tagging to the region between α2 and β4 (insertion site 1) could enable the incorporation of cargo proteins into the shell.

To generate additional inward-facing insertion sites, we created a circularly permuted CsoS1A variant (CsoS1A^P^) by relocating the C-terminal region to the N-terminus (Figure 1c,d). This approach has been employed to successfully invert the sidedness of the N- and C-terminal residues of a BMC hexamer.^36,37^ Gly72 was selected as the site for the permutation, as it is located at the concave surface of CsoS1A and is expected to have minimal effects on the oligomerization of CsoS1A (Figure S1a). The C-terminal segment (DGLVAAHIIARVHSEVENILPK) was moved to the N-terminus with a flexible (Gly-Ser)2 linker connecting the modified N-terminus and the original N-terminus, resulting in the inward-facing N- (insertion site 3) and C-termini (insertion site 4) of CsoS1A^P^ (Figure 1d). The design of CsoS1A^P^ referred to the circular permutation of PduA,^37^ a paralog in Pdu BMCs, in which the last 4 amino acids forming a random coil structure were deleted (Figure S1a).

To assess the effects of permuted CsoS1A^P^ on shell assembly, we generated a *cso-2′* operon (Figures S1a and S2b), which contains the genes encoding α-CB shell proteins (*csoS2*, *csoS4AB*, *csoS1CB*, *csoS1D*) and CsoS1A^P^. Expression of the *cso-2′* operon resulted in the production of polyhedral shell structures in *E. coli*, as determined by thin-section electron microscopy (EM) (Figure S1b). The average diameter of purified *cso-2′* shells enriched in the 20% sucrose fraction was ∼90 nm (Figure S1c), comparable to the purified *cso-2* shells from the same sucrose fraction (∼97 nm).^9^ SDS–PAGE confirmed the presence of the shell proteins CsoS1A^P^, CsoS1C, and CsoS1B, as well as the linker proteins CsoS2A and CsoS2B (Figure S1d). These results demonstrate that the circular permutation of CsoS1A has negligible effects on shell assembly.

Using the CC^A^ peptide as an example, we further examined the structures of CsoS1A and CsoS1A^P^ hexamers fused with AP at the four insertion sites using AlphaFold prediction (Figure 1e). Despite the low confidence in predicting the structure of CC^A^ peptides within CC^A^-fused CsoS1A or CsoS1A^P^ hexamers, the orientation of different insertion sites is distinctly discernible (Figure S3). The CC^A^ peptide fused at insertion site 2 is located on the concave side of the CsoS1A hexamer, whereas the fusion of the CC^A^ peptide at the other three insertion sites results in a convex-facing tag. Importantly, the AlphaFold prediction revealed that the assembly of the CsoS1A hexamer is not impeded by the insertion of CC^A^ at any of the insertion sites. Using these four insertion sites in WT CsoS1A and CsoS1A^P^, we generated a total of 16 different shell constructs, in which CsoS1A or CsoS1A^P^ were fused with the ST/SC system or Coiled-coil system. These shell constructs were employed to target cargos of interest that are fused with their cognate partners (Figure S2).

### Effects of AP Incorporation on Shell Assembly

**Figure 2:**
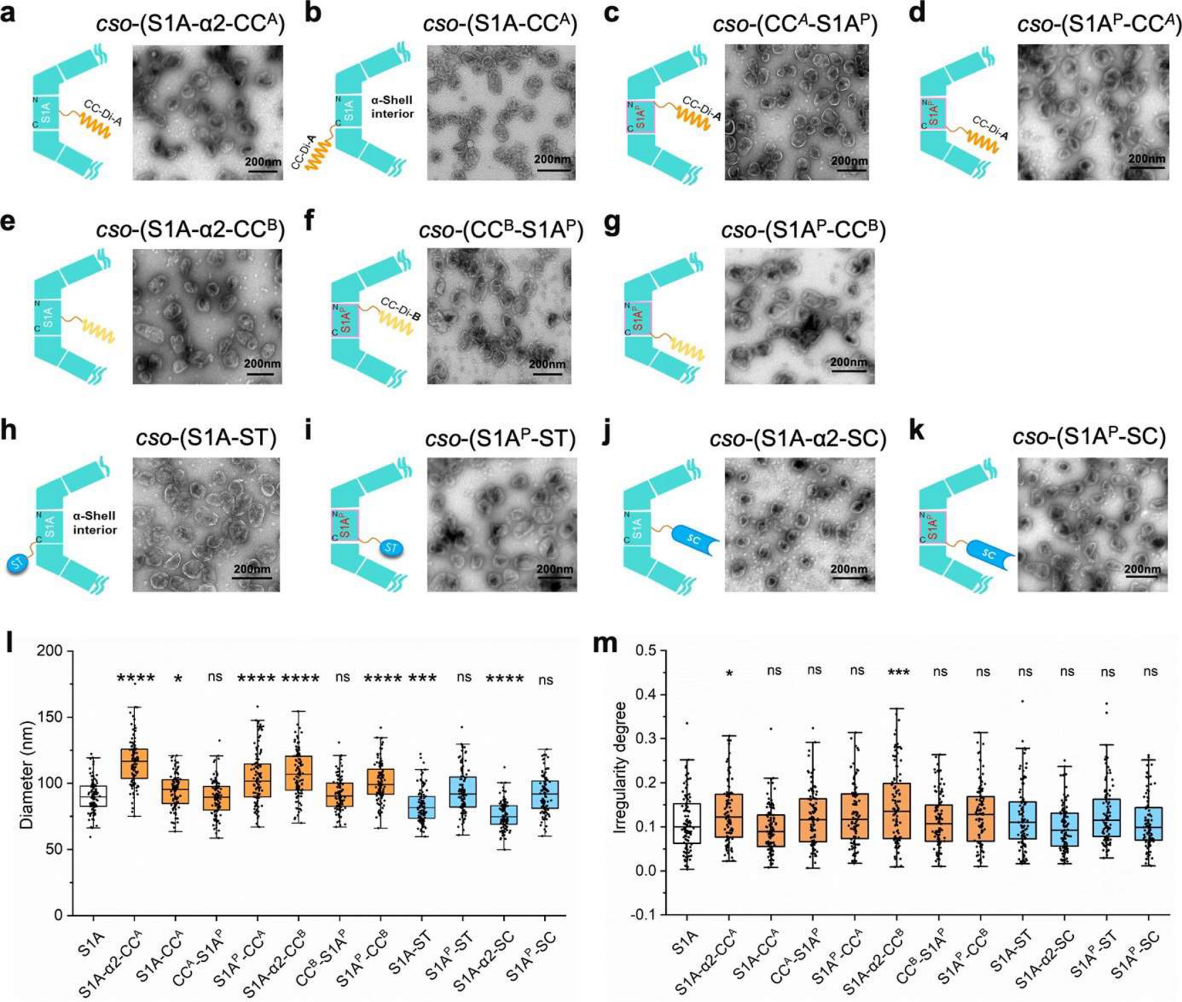
Distinct AP insertions have no noticeable negative effects on shell assembly. (a) EM images of purified AP inserted α-CB shells. Purified α-CB shell with CC^A^ fused at insertion site 1; (b) CC^A^ fused at insertion site 2; (c) CC^A^ fused at insertion site 3; (d) CC^A^ fused at insertion site 4; (e) CC^B^ fused at insertion site 1; (f) CC^B^ fused at insertion site 3; (g) CC^B^ fused at insertion site 4; (h) ST fused at insertion site 2; (i) ST fused at insertion site 4; (j) SC fused at insertion site 1; (k) SC fused at insertion site 4. Scale bar: 200 nm. (l) Size comparison of the 11 types of ST/SC or Coiled-coil system-functionalized shells and *cso-2* shells. The diameter of each polyhedral shell particle was determined by averaging three measurements obtained by drawing diagonals from various angles of the shell. (m) Irregularity comparison of the 11 types of ST/SC or Coiled-coil system-functionalized shells and *cso-2* shells. The irregularity degree was determined by calculating the ratio of the standard deviation to the average of three diagonal measurements for each shell. *, 0.01 ≤ *p* ≤ 0.05; ***, 0.0001 ≤ *p* ≤ 0.001; ****, *p* ≤ 0.0001; ns, no significance (*n* = 100, two-tailed unpaired *t* test).

To examine the effects of AP tagging at individual insertion sites of CsoS1A or CsoS1A^P^ on the assembly of α-CB shells, we expressed and analyzed these *de novo*-designed shell constructs in *E. coli* (Figure S2), in comparison with the α-CB shells using CsoS2-C for cargo encapsulation. Among the 16 constructs, 11 types of AP insertions, including 7 types of CC^A^/CC^B^ insertions (CC^A^ fused at insertion sites 1, 2, 3, 4, CC^B^ fused at insertion sites 1, 3, 4) and 4 types of ST/SC insertions (ST appended at insertion sites 2, 4, SC attached to insertion sites 1, 4), could result in shell formation. Negative-staining EM revealed that stable shell structures with diameters of 76–116 nm were produced by these 11 shell constructs (Figure 2a–i). The irregularity degree of shells with either CC^A^ or CC^B^ fused at insertion site 1 was relatively higher than WT shells, whereas the other AP fused shells exhibited comparable structural heterogeneity as WT shells (Figure 2m). SDS–PAGE analysis indicated the presence of CsoS1C, CsoS1B, CsoS2A, and CsoS2B, as well as AP-inserted CsoS1A or CsoS1A^P^ (Figure S4a–k). These results indicated that these 11 types of AP insertions to CsoS1A/CsoS1A^P^ have no discernible effects on shell assembly and AP-fused CsoS1A/CsoS1A^P^ can be incorporated into the shell.

Moreover, tagging CsoS1A allowed us to differentiate the two highly homologous major shell proteins, CsoS1A and CsoS1C, which differ by only two amino acids out of 98, and determine their ratios in the α-CB shells. Intriguingly, the relative ratio of CsoS1A to CsoS1C varies within a range of 0.15:1–1.24:1 among different AP-inserted shells (Figure S4i), suggesting the reductant functions of CsoS1A and CsoS1C in shell assembly. Notably, only when AP was inserted at the C-terminus of CsoS1A (insertion site 2 at the concave side), the CsoS1A content was significantly higher than that of CsoS1C (ratio >1:1), suggesting that AP tagging at the outward-facing side has less effects on the incorporation of CsoS1A-AP into the α-CB shell than at the inward-facing side. More importantly, the higher content of AP-targeted CsoS1A in the shell suggests a greater cargo-loading capacity.

In contrast, the other five types of AP-inserted shell constructs, including *cso*-(S1A-CC^B^), *cso*-(S1A-SC), *cso*-(S1A-α2-ST), *cso*-(ST-S1A^P^), and *cso*-(SC-S1A^P^) (Figures S5 and S6), failed to mediate shell formation. To elucidate how these insertions impeded shell assembly, we collected the supernatant and pellet after each centrifugation during the shell purification process for SDS–PAGE analysis (Figure S5a,b). A large amount of CsoS1A fused with CC^B^ at the C-terminus (S1A-CC^B^) and other shell proteins were pelleted with cell debris by 10 000*g* centrifugation; after the supernatant was further centrifugated at 50 000*g*, no S1A-CC^B^ was detected in the pellet where the assembled α-CB shells were typically found. This result suggests that S1A-CC^B^ tends to form protein aggregates and co-precipitate with cell debris (Figure S5a). Similarly, a small amount of CsoS1A with SC inserted at the C-terminus (S1A-SC) was detected in the pellet after 50 000*g* centrifugation, whereas the majority of S1A-SC tended to form aggregates and were present in the pellet after 10 000*g* centrifugation (Figure S5b), indicating the SC fusion impeded shell formation. We speculate that the interactions between AP fused at these specific sites of CsoS1A led to the aggregation of nonassembled shell proteins.

To test this hypothesis, we used enhanced green fluorescent protein (GFP) as a reporter to determine the aggregation status of AP in *E. coli*. Confocal images revealed that GFP with CC^A^ fused at either the N- or C-terminus exhibited evenly distributed fluorescent signals, suggesting that CC^A^ did not self-aggregate, whereas GFP with CC^B^ fused at the N-terminus exhibited large foci at the cell poles (Figure S5c). These results indicate the self-association of CC^B^, consistent with previous observations of the CC^B^-based fusion.^37^ Furthermore, cells producing GFP with CC^B^ at the C-terminus show relatively weak foci at the end of the cell (Figure S5c), indicating that the degree of self-association varied depending on the insertion site where CC^B^ was appended. Similarly, ST- or SC-based fusion on the N- or C-terminus of GFP led to distinct fluorescence distributions (Figure S5d). Interestingly, fusion of SC at the C-terminus of EutM, a structural analog of CsoS1A in ethanolamine-utilization BMCs, did not have significant effects on the assembly of EutM scaffolds,^39^ suggesting the different effects of the exogenous SC tag on shell protein structure and assembly.

When ST was fused at the region between α2 and β4 of CsoS1A (insertion site 1, Figure 1c), the resulting CsoS1A-α2-ST was detected in the pellet after 50 000*g* centrifugation, while the majority of CsoS1A-α2-ST was found in the supernatant. However, after sucrose gradient centrifugation at 105 000*g*, most of the CsoS1A-α2-ST was found in the top layer (Figure S6a). These results suggest that CsoS1A-α2-ST may be involved in shell assembly, but the CsoS1A-α2-ST-incorporated shells appear to be unstable and tend to disassemble during centrifugation, resulting in a large amount of free CsoS1A-α2-ST and CsoS1C being released from the shell assemblies (Figure S6a). For the circularly permuted CsoS1A^P^, when ST or SC was fused to the N-terminus of CsoS1A^P^, neither of the tagged CsoS1A^P^ was detected in whole cell lysates by SDS–PAGE and immunoblot analysis (Figure S6b). It is presumed that the insertion of ST and SC at the N-terminus of CsoS1A^P^ may affect the protein expression or the solubility of fused proteins (Figure S6b). Interestingly, fusion of CC^A^/CC^B^ at the same insertion site resulted in the formation of stable shells (Figure 2c,f). These observations highlight the distinct effects of different APs at the same insertion site on shell assembly. Overall, our findings indicate that the location of AP insertion on CsoS1A is a key factor in determining the degree of protein aggregation. This could be an important consideration in rational design of AP fusion for protein/enzyme immobilization.

### AP Mediates Colocalization of the Shell and Cargos

**Figure 3:**
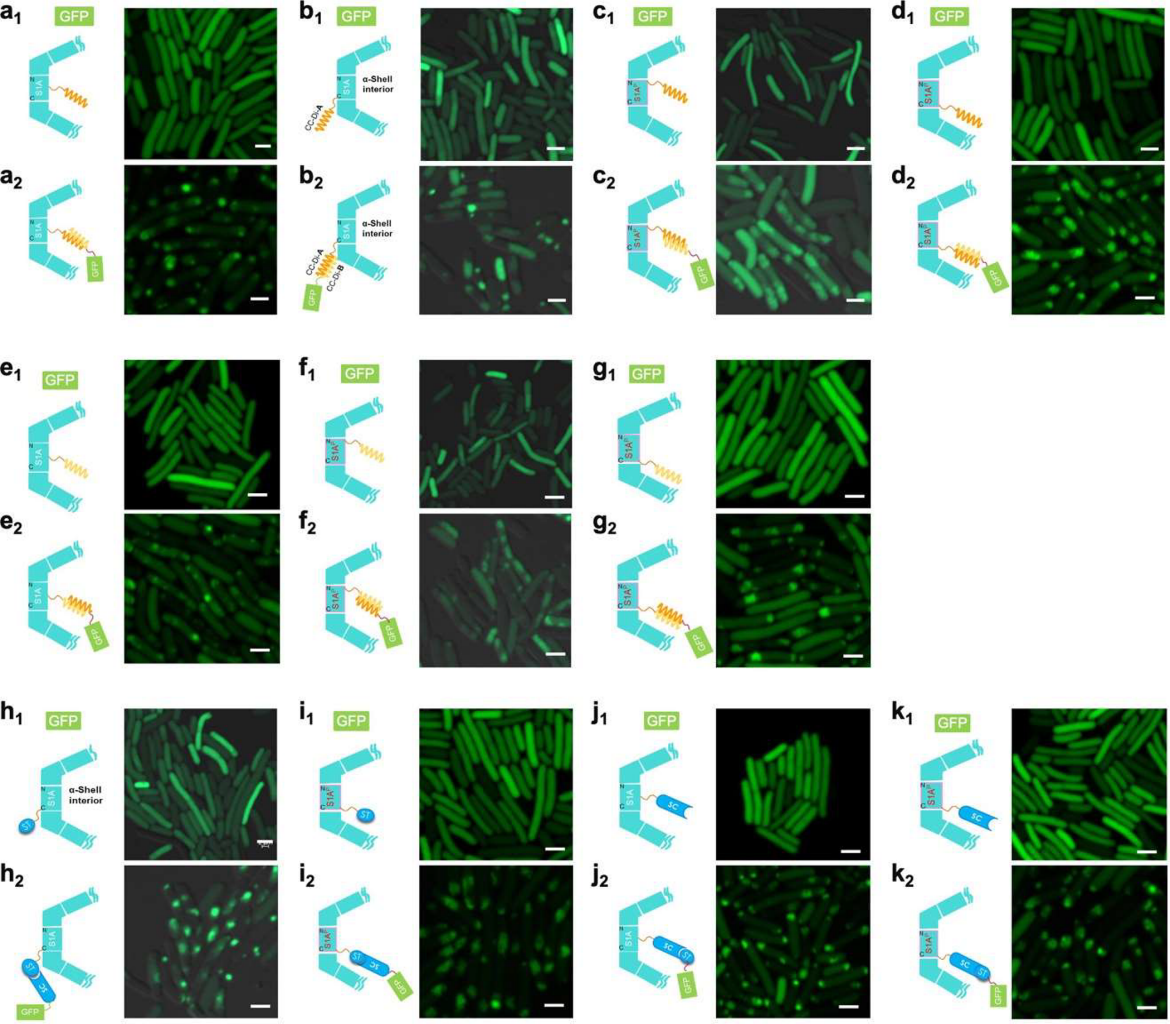
Colocalization analysis of GFP and AP-inserted shells. Confocal images of *E. coli* cells coexpressing free GFP and AP-inserted shells (a_1_–k_1_) and cells coexpressing GFP-AP and cognate AP-inserted shells (a_2_–k_2_) indicating different types of AP could mediate GFP on the shell surface or incorporation of GFP into the shell. (a_1_, a_2_) Free GFP or GFP-CC^B^ coexpressed with S1A-α2-CC^A^ mediated shells. (b_1_, b_2_) Free GFP or GFP-CC^B^ coexpressed with S1A-CC^A^ mediated shells. (c_**1**_, c_2_) Free GFP or GFP-CC^B^ coexpressed with CC^A^-S1A^P^ mediated shells. (d_**1**_, d_2_) Free GFP or GFP-CC^B^ coexpressed with S1A^P^-CC^A^ mediated shells;. (e_1_, e_2_) Free GFP or GFP-CC^A^ coexpressed with S1A^P^-α2-CC^B^ mediated shells. (f_1_, f_2_) Free GFP or GFP-CC^A^ coexpressed with CC^B^-S1A^P^-mediated shells. (g_**1**_, g_2_) Free GFP or GFP-CC^A^ coexpressed with S1A^P^-CC^B^ mediated shells. (h_1_, h_2_) Free GFP or GFP-SC coexpressed with S1A-ST mediated shells. (i_1_, i_2_) Free GFP or GFP-SC coexpressed with S1A^P^-ST mediated shells. (j_1_, j_2_) Free GFP or GFP-ST coexpressed with S1A^P^-α2-SC mediated shells. (*k*_1_,*k*_2_) Free GFP or GFP-ST coexpressed with S1A^P^-SC mediated shells. Scale bar: 2 μm. The samples of GFP-APs coexpressed with α-CB shells that do not have the partner AP were not included as controls, as our data indicate that the GFP-APs did not form detectable aggregates that could affect the analysis (see Figures S4 and S6).

To determine the capacities of the 11 types of AP-fused CsoS1A/CsoS1A^P^ that led to shell formation in cargo targeting, we generated 4 plasmids that express GFP with its C-terminus fused with different APs: CC^A^, CC^B^, ST, or SC. These plasmids were then coexpressed with the α-CB shells that were incorporated with the corresponding cognate peptides to enable AP interactions. Confocal images showed that unlike free GFP that exhibited a diffusive fluorescence signal throughout the cell in the presence of AP-fused shells (Figure 3a_1_–k_1_), GFP labeled with the cognate AP partners exhibited dispersed fluorescent foci (Figure 3a_2_–k_2_), suggesting the colocalization of targeted GFP and the shell mediated by AP interactions. Additionally, the punctate fluorescence signal relative to the cytoplasmic fluorescence varied among the constructs, suggesting distinct cargo-loading capacities of different types of AP-incorporated systems.

**Figure 4:**
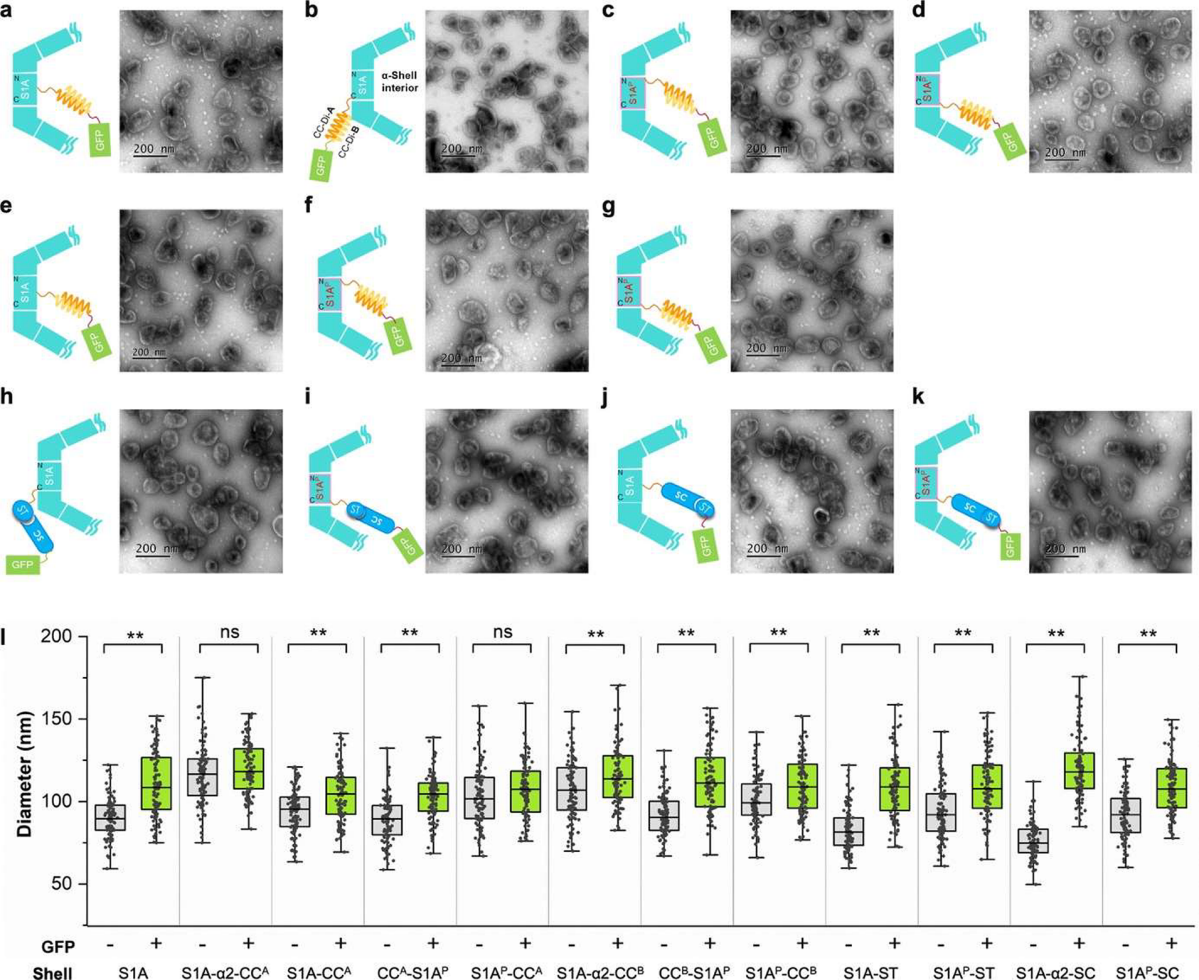
Effects of GFP loading on the structures of α-CB shells. EM images of 11 types of GFP loaded shells. (a) S1A-α2-CC^A^ mediated GFP-loaded shells; (b) S1A-CC^A^ mediated GFP-loaded shells; (c) CC^A^-S1A^P^ mediated GFP-loaded shells; (d) S1A^P^-CC^A^ mediated GFP-loaded shells; (e) S1A^P^-α2-CC^B^ mediated GFP-loaded shells; (f) CC^B^-S1A^P^-mediated GFP-loaded shells; (g) S1A^P^-CC^B^ mediated GFP-loaded shells; (h) S1A-ST mediated GFP-loaded shells; (i) S1A^P^-ST mediated GFP-loaded shells; (j) S1A-α2-SC mediated GFP-loaded shells; (k) S1A^P^-SC mediated GFP-loaded shells. (l) Size comparison of the ST/SC or Coiled-coil system-functionalized shells with or without GFP cargo, as well as the size of *cso-2* shells with or without GFP. **, 0.001 ≤ *p* ≤ 0.01; ns, no significance (*n* = 100, two-tailed unpaired *t* test).

Furthermore, these GFP-loaded shells were purified from *E. coli* using sucrose gradient ultracentrifugation. Immunoblot analysis revealed that the Shell-GFP assemblies were enriched in the 20% or 30% sucrose fractions; in contrast, free GFP-AP was only present in the top layer of the sucrose gradient (Figure S7). These results further confirmed that these APs could mediate GFP incorporation to the α-CB shells. Interestingly, EM showed that the purified Shell-GFP assemblies were mostly larger than empty AP-fused shells collected from the 30% sucrose fractions (Figure 4a–l), suggesting that the integration of GFP may result in enlargement of the shell structure. Moreover, the GFP-loaded shells appear to have a thicker shell compared to empty AP-inserted shells (Figures 2a–k and 4a–k), which is consistent with previous findings,^36^ presumably owing to the presence of GFP on the outer or inner surfaces of α-CB shells.

### Comparison of the Cargo-Loading Capacities of Different AP Systems

To examine the cargo-loading capacities of different AP-based shells, we purified the 11 AP-based Shell-GFP assemblies along with the CsoS2-C-mediated Shell-GFP assemblies. Since free GFP-AP were not present in the 10–50% sucrose fractions (Figure S7), we determined the ratios of the content of GFP and CsoS1 (including AP-fused CsoS1A, WT CsoS1B and CsoS1C) in individual sucrose fractions through immunoblot analysis using anti-GFP and anti-CsoS1 antibodies, which are used as an indicator for the GFP-loading capacity of distinct AP-modified shells.

**Figure 5:**
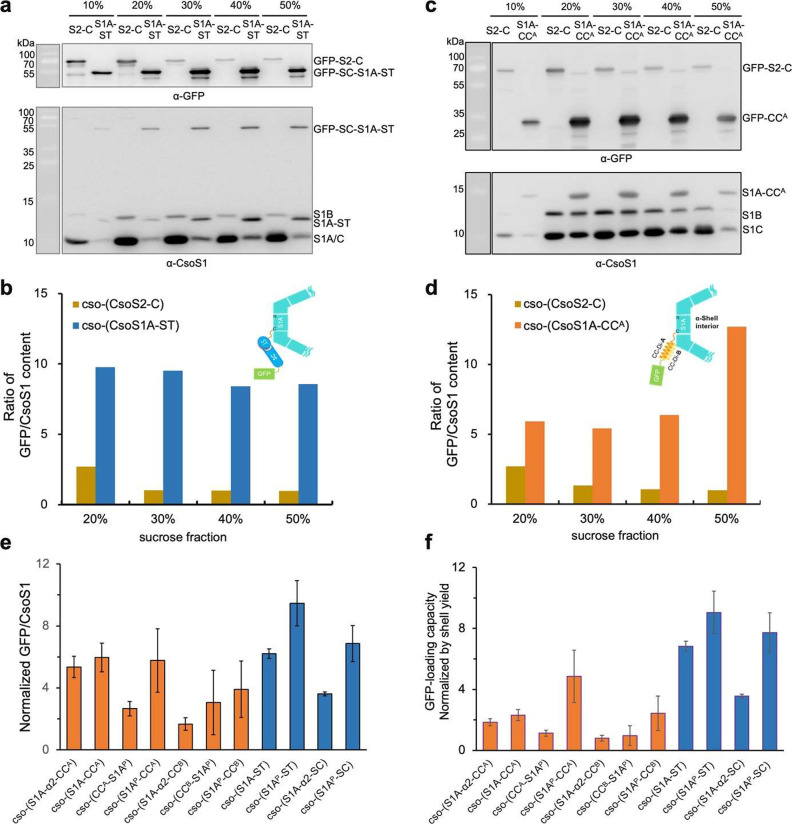
Cargo-loading capacities of the AP-insertion systems. (a) Immunoblot analysis of samples purified by sucrose gradient centrifugation from *E. coli* cells that express CsoS2-C mediated Shell-GFP assemblies and S1A-ST mediated Shell-GFP assemblies. (b) Ratios of GFP and CsoS1 content in individual sucrose fractions, quantified based on the band densities in (a). The ratios of GFP and CsoS1 content in cells producing S1A-ST-mediated Shell-GFP assemblies are significantly higher than those in cells producing CsoS2-C-mediated Shell-GFP assemblies, indicating that the CsoS1A-ST-based system has a greater cargo-loading capacity than that of the CsoS2-C-based cargo-loading system. (c) Immunoblot analysis of sucrose fractions purified from cells expressing CsoS2-C-mediated Shell-GFP assemblies and S1A-CC^A^-mediated Shell-GFP assemblies. (d) Quantification of the ratios of GFP and CsoS1 content in individual sucrose fractions based on the band densities in (c). (e) Ratios of GFP and CsoS1 content of 11 types of Shell-GFP assembles normalized to that of CsoS2-C-based Shell-GFP assemblies, as indicators of the GFP-loading capacity of different AP-based cargo-loading systems. Data were collected from three independent immunoblot results. (f) GFP-loading capacity normalized by the shell yield in the 11 types of cells expressing Shell-GFP assembles. Data were collected from three independent immunoblot results.

CsoS1 proteins and covalently bound GFP-SC-S1A-ST were mainly distributed in the 20–50% sucrose fractions, and the GFP/CsoS1 ratio of S1A-ST-mediated Shell-GFP assemblies in each sucrose fraction was significantly higher than that of CsoS2-C-mediated Shell-GFP assemblies (Figure 5a,b). Similarly, the other three types of ST/SC-based Shell-GFP assemblies, as well as the S1A-CC^A^-based Shell-GFP assemblies and the other 6 types of Coiled-coil-mediated Shell-GFP assemblies, also exhibited greater GFP/CsoS1 ratios than that of CsoS2-C-mediated Shell-GFP assemblies in each sucrose fraction (Figure 5c,d and Figures S8 and S9). These results reveal that both the ST/SC-based and Coiled-coil-based cargo-loading systems are more efficient in recruiting cargo proteins than the endogenous EP, CsoS2-C.

To further compare the cargo-loading capacities of the ST/SC system and the Coiled-coil system, the average ratios of GFP and CsoS1 content in the 20–50% sucrose fractions of AP-based systems were normalized by those of the CsoS2-C based system in the corresponding sucrose fractions. Among the 11 different Shell-GFP assemblies, the S1A^P^-ST-based system, in which ST is located on the convex side of CsoS1A^P^ allowing for encapsulation of GFP-SC into the shell, showed the highest GFP-loading capacity (Figure 5e), ∼9.5-fold greater than that of the CsoS2-C system. In contrast, the S1A-α2-CC^B^-based system appeared to be the least effective cargo-immobilization system, but it still exhibited a 1.7-fold increase in the GFP-loading capacity compared with the CsoS2-C system. Among the Coiled-coil-based systems, the S1A-CC^A^-based Shell-GFP assemblies (*cso*-(S1A-CC^A^) are the most efficient in targeting cargos, with a ∼6-fold increase in the GFP-loading capacity than the S2-C system, comparable to the ST/SC-based system with ST fused at the same site (insertion site 2) on the concave side of WT CsoS1A (*cso*-(S1A-ST)) (Figure 5e). It is noteworthy that the CC^A^-based Shell-GFP assemblies exhibited a higher cargo-loading capacity than the CC^B^-based Shell-GFP assemblies with CC^B^ fused at the same position. Consistently, the ST-fused system also exhibited a greater cargo-loading capacity than the SC-based system with SC inserted at the same position. This discrepancy in cargo-loading capacity between AP pairs might be attributed to their distinct chemical or structural properties, such as surface acidity or basicity, molecular weight, and secondary structure. The different surface properties of CC^A^ and CC^B^, such as their acidity or basicity, might result in various levels of compatibility with shell proteins. This, in turn, may have distinct effects on the shell assembly and cargo loading. For the SC-based system, the large SC tag may result in greater steric effects and clashes with other shell components, thereby leading to a lower cargo-loading capacity than the ST-based system.

Based on the GFP-SC-CsoS1A^P^-ST/CsoS1 ratio in the 30% sucrose fraction of CsoS1A^P^-ST-based Shell-GFP assemblies, approximately 7.3% of the total CsoS1 per shell was occupied by GFP-AP (Figure S8b). As the stoichiometric quantification data show that recombinant α-CB contains 6476 copies of CsoS1 monomers,^17^ we roughly estimated that approximately 473 GFP-AP molecules were encapsulated in each CsoS1A^P^-ST shell, which is significantly more efficient than a *Haliangium ochraceum* shell-based cargo-loading system (up to 80 GFP molecules per shell).^36^

Moreover, the yield of α-CB shells produced in *E. coli* cells is another important factor for evaluating the cargo-loading capacities of the shell assemblies. To achieve this, cell lysates containing equal amounts of proteins from different types of Shell-GFP assemblies were subjected to 50 000*g* centrifugation, and the resulting pellets containing the Shell-GFP assemblies were examined by immunoblot analysis using an anti-CsoS1 antibody. We found that cells expressing AP-based shell assemblies exhibited various degrees of reduction in shell yield, particularly for the cells expressing Coiled-coil-fused Shell-GFP assemblies (Figure S10). This observation suggests that the Coiled-coil fusion has more notable effects on the yield of shells or shell protein expression than the ST/SC systems, possibly due to the self-aggregation of the CC^B^ peptides, which may interfere with shell assembly or cargo loading. Intriguingly, taking the shell yield into consideration, the cargo-loading capacity varies between AP pairs. the S1A^P^-ST-based system still exhibited superior GFP-loading capacity than other AP-fused Shell-GFP assemblies, with a 9.3-fold increase in the GFP-loading capacity than the CsoS2-C-based system (Figure 5f). The ST-based system has a higher cargo-loading capacity than the SC-based system, consistent with previous findings.^43^ The S1A-CC^A^-based system, in which CC^A^ is exposed on the concave side for immobilizing GFP-CC^B^ on the outer surface of the shell, possesses a greater cargo-loading capacity than other Coiled-coil based systems (Figure 5f). In contrast, fusions at the region between α2 and β4 of CsoS1A, including S1A-α2-CC^A^, S1A-α2-CC^B^ and S1A-α2-ST-based systems, resulted in a significant reduction in the yield of shells and relatively low cargo-loading capacity.

## Conclusion

In this study, we developed innovative ways to create α-CB shell-based nanocages with customized sites for cargo recruitment and systematically evaluated their assembly and cargo-loading efficiencies. We established site-directed cargo-loading systems that are capable of recruiting heterologous cargos to the specific sites on the outer or inner shell surfaces of the α-CB shell-based nanocages. Through *de novo* design and synthetic engineering, we generated 16 different types of recombinant α-CB shells, in which the exogenous ST/SC and Coiled-coil systems were fused at four distinct insertion sites of WT CsoS1A and circularly permuted CsoS1A^P^, and performed a comprehensive assessment of the cargo-loading capacities of these shell assemblies. Our results demonstrate that 11 types of ST/SC and CC^A^/CC^B^ fusions on CsoS1A or CsoS1A^P^ could lead to the formation of stable shell structures with a diameter of 90–120 nm. We further reveal that these custom-engineered shells exhibited improved capacities of recruiting GFP as non-native cargos into or onto the shell structures. Intriguingly, both the ST/SC and Coiled-coil systems exhibited superior cargo-recruitment capacities when compared to the endogenous encapsulation peptide CsoS2-C, while the ST/SC system exhibits advantages over the Coiled-coil system. Furthermore, the diverse cargo-loading capacities indicate the versatility and fine-tunability of cargo loading and capture of these generated nanocages, which allow them to hold significant potential in diverse biotechnological and biomedical applications such as enhancing the catalytic performance of encapsulated cargo enzymes within the shell or facilitating molecule delivery by binding specific molecules or drugs on the outer surface of the shell. Our findings provide insights into the encapsulation principles of CBs and offer strategies for engineering designable CB shell-based nanocages to enhance cargo capture and encapsulation as well as protection and delivery of molecules, such as enzymes (including hydrogenases for biofuel production^9,31^), DNA, and RNA. It also highlights the great potential to precisely manipulate cargo–shell interactions and the electrostatic properties of the shell outer and inner surfaces, such as through computational design, genetic engineering, and adjusting pH or ion concentrations.

## Materials and Methods

### Generation of Constructs

All connections between genes and linearized vectors were achieved by Gibson assembly (Gibson assembly kit, New England BioLabs, U.K.). The *cso-2′* operon was generated by replacing wild type *csoS1A* gene in the *cso-2* operon derived from *Halothiobacillus neapolitanus*(9) with synthesized circularly permuted *csoS1A*^*P*^ gene. For the construction of *cso* operons with AP inserted at the insertion site 1, the nucleotide sequence encoding CC^A^, CC^B^, SpyTag (ST) and SpyCatcher (SC) flanked by GSGGSG linker was inserted at the insertion site 1 of the *csoS1A* gene in the *cso-2* vector to generate *cso* operons expressing *cso*-(S1A-α2-CC^A^), *cso*-(S1A-α2-CC^B^), *cso*-(S1A-α2-ST) and *cso*-(S1A-α2-SC), respectively. For the construction of *cso* operons with AP inserted at the insertion site 2, the nucleotide sequence encoding CC^A^, CC^B^, ST, and SC was fused to the C-terminus of CsoS1A in the *cso-2* vector with a 18 amino acid linker composed of GSGSGSHHHHHHGSGGSG linker, resulting in operons expressing *cso*-(S1A-CC^A^), *cso*-(S1A-CC^B^), *cso*-(CsoS1A-ST), and *cso*-(CsoS1A-SC), respectively. For the construction of *cso* operons with AP inserted at the insertion site 3, the nucleotide sequence encoding CC^A^, CC^B^, ST, and SC was attached to the N-terminus of CsoS1A^P^ in the *cso-2’* vector with a GSGSGSHHHHHHGSGGSG linker to generate *cso* operons producing *cso*-(CC^A^-S1A^P^), *cso*-(CC^B^-S1A^P^), *cso*-(ST-S1A^P^), and *cso*-(SC-S1A^P^), respectively. For the construction of *cso* operons with AP inserted at the insertion site 4, the nucleotide sequence encoding CC^A^, CC^B^, ST, and SC was fused to the C-terminus of CsoS1A in the *cso-2* vector with a 18 amino acid linker composed of GSGSGSHHHHHHGSGGSG linker, resulting in operons expressing *cso*-(S1A^P^-CC^A^), *cso*-(S1A^P^-CC^B^), *cso*-(S1A^P^-ST), and *cso*-(S1A^P^-SC), respectively.

The enhanced *gfp* gene was cloned into pCDFDueT-1 linearized by NcoI and NotI under the control of a pTrc promoter to generate the pCDF-Trc-GFP vector. The *gfp* gene, in frame with the nucleotide sequence encoding 6× poly-histidine tag, with the nucleotide sequence of four types of AP fused either at the N- or C-terminus under the control of a pTrc promoter was cloned into pCDFDueT-1 to create pCDF-GFP-CC^A^, pCDF-GFP-CC^B^, pCDF-GFP-ST, pCDF-GFP-SC, pCDF-CC^A^-GFP, pCDF-CC^B^-GFP, pCDF-ST-GFP, and pCDF-SC-GFP, respectively. All of these constructs were verified by PCR and DNA sequencing and transformed into *E. coli* DH5α and BW25113 cells.

### Expression and Isolation of α-CB Shells

*E. coli* strains containing the 16 types of AP-inserted *cso* vectors were cultivated at 37 °C in Lysogeny Broth (LB) medium containing 100 μg mL^–1^ ampicillin. The expression of these vectors was induced by l-Arabinose (1 mM, final concentration) once the cells reached an early log phase (OD_600_ = 0.6). Cells were grown at 25 °C for 16 h with constant shaking and then were harvested by centrifugation at 4000*g* for 10 min. The cell pellets were washed with TEMB buffer (10 mM Tris-HCl, pH = 8.0, 1 mM EDTA, 10 mM MgCl_2_, 20 mM NaHCO_3_) and resuspended in TEMB buffer supplemented with 10% (v/v) CelLytic B cell lysis reagent (Sigma-Aldrich) and 1% protein inhibitor cocktail (100×) (Sigma-Aldrich). The cell suspensions were lysed by sonication, and cell debris was removed by centrifugation, followed by centrifugation at 50 000*g* to enrich α-CB shells. The pellets were resuspended in TEMB buffer and then loaded onto sucrose gradients (10–50%, w/v) followed by ultracentrifugation (BeckMan, XL100K ultracentrifuge) at 105 000*g* for 30 min. Each sucrose fractions were collected and stored at 4 °C.

### Expression and Isolation of GFP-Loaded α-CB Shells

*E. coli* strains coexpressing the AP inserted shell and GFP fused with the corresponding partner AP peptide were cultivated at 37 °C in lysogeny broth (LB) medium containing 100 μg mL^–1^ ampicillin and 50 μg mL^–1^ spectinomycin. The GFP-AP expression was induced by the addition of 0.25 mM IPTG at an OD600 = 0.6. After 4 h of induction of the GFP-AP expression, the shell expression was induced by 1 mM l-arabinose, and cells were then grown at 25 °C for 16 h. The isolation of GFP-incorporated shells was purified following the protocol described above for the empty shell purification.

### SDS–PAGE and Immunoblot Analysis

SDS–PAGE and immunoblot examination were performed following the procedure described previously.^53−55^ Briefly, 20 or 40 μg of total protein was loaded into each well for immunoblotting and Coomassie staining, respectively. Immunoblot analysis was performed using primary mouse monoclonal anti-His (Invitrogen, catalog no. MA1-135 dilution 1:3000), rabbit polyclonal anti-CsoS1 (Agrisera, catalog no. AS142760, dilution 1:3000), and horseradish peroxidase-conjugated goat anti-mouse IgG secondary antibody (Agrisera, catalog no. AS111772, dilution 1:10 000) and anti-rabbit IgG secondary antibody (Agrisera, catalog no. AS09602, dilution 1:10 000). Signals were visualized by using a chemiluminescence kit (Bio-Rad). Immunoblot images were collected by ImageQuant LAS 4000 software, version 1.2.1.119. Immunoblot protein quantification was performed using ImageJ software (version 1.52 h). For each experiment, at least three biological repeats were examined.

### Transmission Electron Microscopy

Thin-section transmission electron microscopy (EM) was performed to visualize the reconstituted shell structures in *E. coli* strains. Isolated shell structures were characterized using negative staining EM.^30^ Images were recorded using an FEI Tecnai G2 Spirit BioTWIN transmission electron microscope equipped with a Gatan Rio 16 camera. Image analysis was carried out by using ImageJ software. The shell diameter data was randomly collected from 100 shell particles on EM images. The diameter of each polyhedral shell particle was measured by drawing diagonals three times from various angles, all intersecting at the same center point, using ImageJ software, and the resulting measurements were then averaged. The irregularity degree was determined by calculating the ratio of the standard deviation to the average of three diagonal measurements for each shell (Figure 2m).

### Confocal Microscopy

Overnight induced *E. coli* cells were immobilized by drying a droplet of cell suspension onto LB agar pads as described previously.^53^ Blocks of agar with the cells absorbed onto the surface were covered with a coverslip and placed under the microscope. Laser-scanning confocal fluorescence microscopy imaging was performed on a Zeiss LSM780 confocal microscope with a 63×/1.4 NA oil immersion objective with an excitation wavelength at 488 nm and emission at 520 nm. Live-cell images were recorded from at least three different cultures. All images were captured with all pixels being below saturation. Image analysis was carried out using ImageJ software.

### Alphafold Prediction Metrics

Structure predictions of the AP-fused CsoS1A or CsoS1A^P^ were performed with AlphaFold2.ipynb (version 1.5.5), following the instructions at the Web site https://colab.research.google.com/github/sokrypton/ColabFold/blob/main/AlphaFold2.ipynb (accessed October 2023). The pLDDT confidence scores for the protein structure models that we predicted by AlphaFold were extracted from the pickle file, from “plddt” array. Prediction was conducted without using template information, and all other settings remained at default configurations. By default, AlphaFold produces five models. We used the one with the highest value of pLDDT for analysis (Figure S3).

### Statistics and Reproducibility

All experiments reported here were performed at least three times independently, and at least three biological repeats were performed for each experiment.

## Supporting Information Available

Figures S1–S10 including additional SDS–PAGE, EM images, protein sequences, Alphafold predictions, cartoon models, confocal images, and Western blotting results (PDF)
