# Supplementary material for: Nanoengineering Carboxysome Shells for Protein Cages with Programmable Cargo Targeting
Source: ACS Nano. 2024 Feb 7;18(10):7473–84. doi: 10.1021/acsnano.3c11559 (PMC10938918; doi:10.1021/acsnano.3c11559)
Supplement: Supplementary file 1 — nn3c11559_si_001.pdf [file nn3c11559_si_001.pdf]

Supporting Information

Nanoengineering carboxysome shells for protein cages with programmable cargo targeting

Tianpei Li[1,2], Ping Chang[2], Weixian Chen[1], Zhaoyang Shi[1], Chunling Xue[1], Gregory F. Dykes[2], Fang Huang[2], Qiang Wang[1], Lu-Ning Liu[2,3*]*

[1] State Key Laboratory of Crop Stress Adaptation and Improvement, School of Life Sciences, Henan University, Kaifeng 475004, China

[2] Institute of Systems, Molecular and Integrative Biology, University of Liverpool, Liverpool L69 7ZB, United Kingdom

[3] MOE Key Laboratory of Evolution and Marine Biodiversity, Frontiers Science Center for Deep Ocean Multispheres and Earth System & College of Marine Life Sciences, Ocean University of China, Qingdao 266003, China

* Correspondence: luning.liu@liverpool.ac.uk (L.-N.L.); wangqiang@henu.edu.cn (Q.W.)

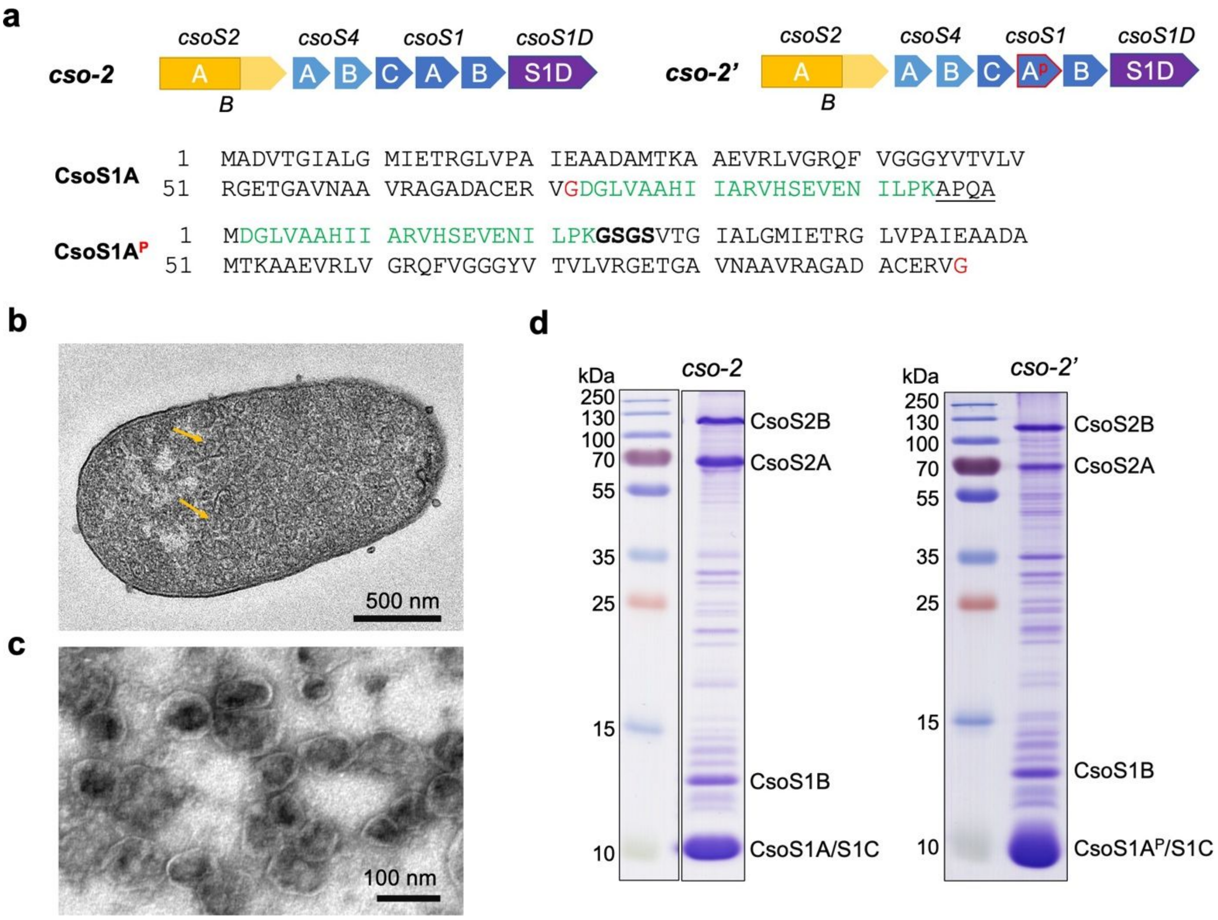

Figure S1. Circular permutation of CsoS1A (CsoS1A^{P}) and characterization of the CsoS1A^{P}-incorporated *cso-2’* shells. (a) Genetic organization of the *cso-2* and *cso-2’* operon and the primary structure of CsoS1A and CsoS1A^{P}. The *cso-2’* operon was generated by replacing the native *csoS1A* gene with circularly permuted *csoS1A^{P}* gene. The C-terminal segment (in green) of CsoS1A was moved to the N-terminus with a flexible GSGS (in bold) linker connecting the modified N-terminus and the original N-terminus to form CsoS1A^{P}. The last four amino acids APQA (underlined) of CsoS1A, which form random coil, were deleted after circular permutation.[37] **(b)** Thin-section electron microscopy (EM) of *E. coli* cells expressing the *cso-2’* operon. Arrows indicate α-CB shell particles discerned in the cell. **(c)** EM of purified *cso-2* shells (left) and *cso-2’* shells (right) collected at the 20% sucrose fraction. **(d)** SDS-PAGE of purified *cso-2’* shells, in comparison to *cso-2* shells characterized previously.[9] Adapted with permission under a Creative Commons [CC BY] License from [9]. Copyright [2020/Li et al.] [Springer Nature/Li et al.].

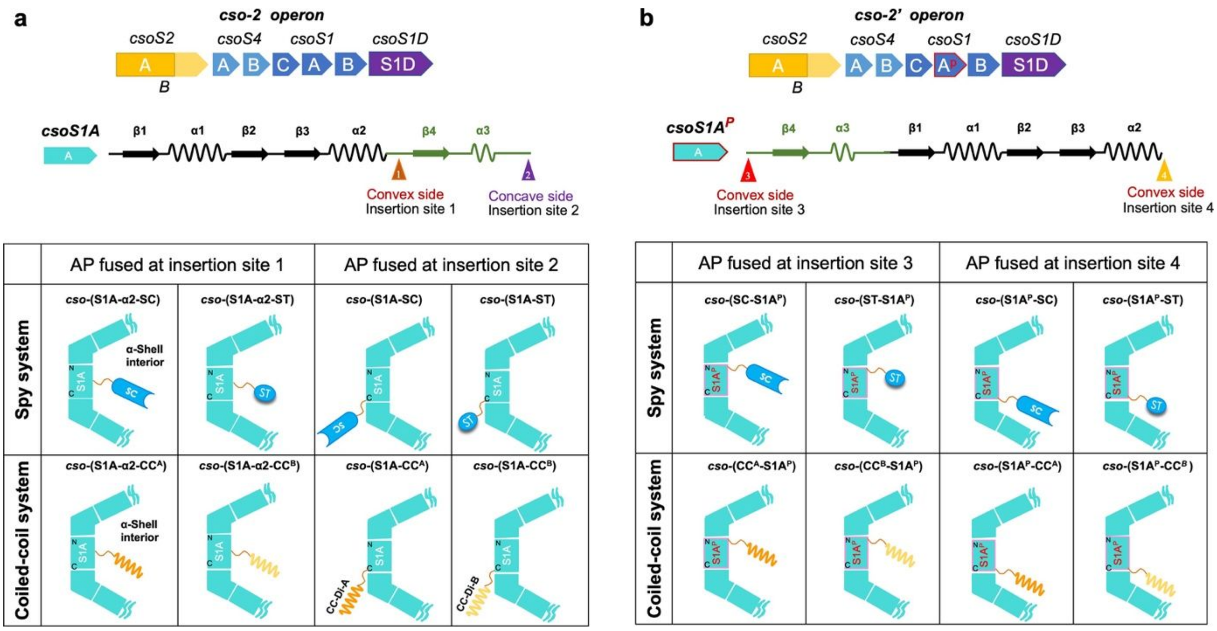

Figure S2. Schematic models of AP-inserted shells. Cartoon models of α-carboxysome shells with ST/SC or CC^A/CC^B fused at different insertion sites either on WT CsoS1A **(a)** or CsoS1A^P **(b)**. ST/SC or CC^A/CC^B fused at the C-terminus of WT CsoS1A located on the concave side of the hexamer and therefore on the outer surface of the shell, while insertion at other three sites of CsoS1A and CsoS1A^P locate within the shell's lumen. The labels of N and C on the CsoS1A module in the cartoon models represent the N- and C-termini of CsoS1A, respectively. The CsoS1A^P module with a red frame represents CsoS1A^P.

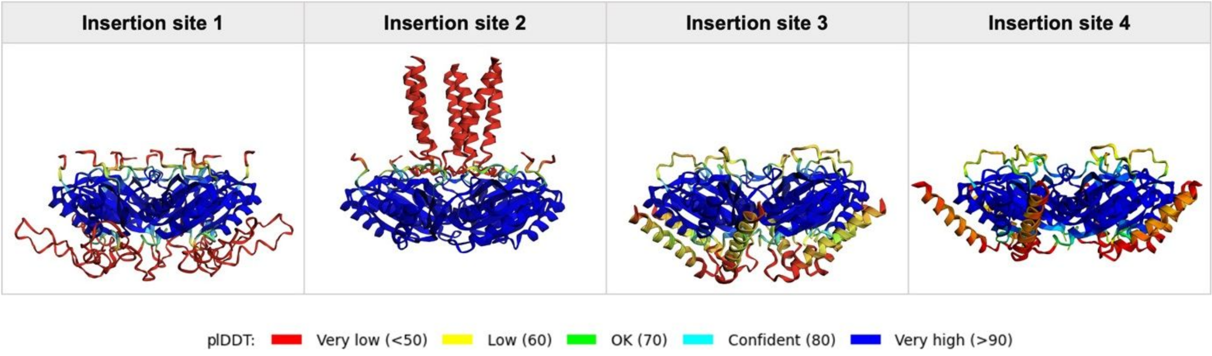

Figure S3. AlphaFold2-predicted structures in Figure 1e colored by model confidence scores (pLDDT). Predicted models of four types of CsoS1A/CsoS1A^{P} hexamers with CC^{A} fused at individual insertion sites. The predicted models are colored according to pLDDT.

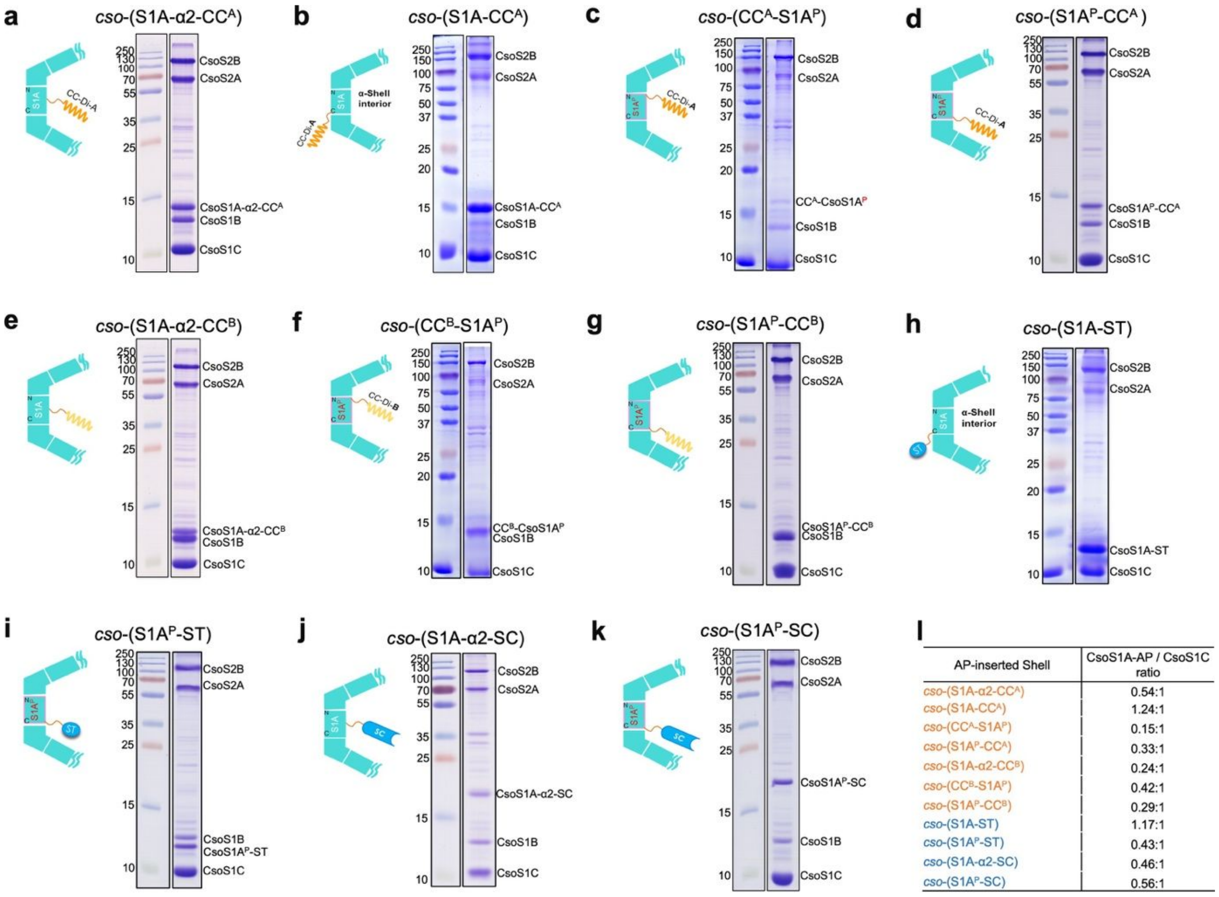

AP-inserted Shell	CsoS1A-AP / CsoS1C ratio
cso-(S1A-α2-CC^A^)	0.54:1
cso-(S1A-CC^A^)	1.24:1
cso-(CC^A^-S1A^P^)	0.15:1
cso-(S1A^P^-CC^A^)	0.33:1
cso-(S1A-α2-CC^B^)	0.24:1
cso-(CC^B^-S1A^P^)	0.42:1
cso-(S1A^P^-CC^B^)	0.29:1
cso-(S1A-ST)	1.17:1
cso-(S1A^P^-ST)	0.43:1
cso-(S1A-α2-SC)	0.46:1
cso-(S1A^P^-SC)	0.56:1

Figure S4. SDS-PAGE of 11 types of purified AP-integrated α-shells. Purified α-carboxysome shell with CC^A fused at the insertion site 1 **(a)**; CC^A fused at the insertion site 2 **(b)**; CC^A fused at the insertion site 3 **(c)**; CC^A fused at the insertion site 4 **(d)**; CC^B fused at the at the insertion site 1 **(e)**; CC^B fused at the at the insertion site 3 **(f)**; CC^B fused at the insertion site 4 **(g)**; ST fused at the insertion site 2 **(h)**; ST fused at the insertion site 4 **(i)**; SC fused at the insertion site 1 **(j)**; SC fused at the insertion site 4 **(k)**. **(l)** The relative ratios of AP-fused CsoS1A (CsoS1A-AP) to CsoS1C varies among different types of AP-inserted shells.

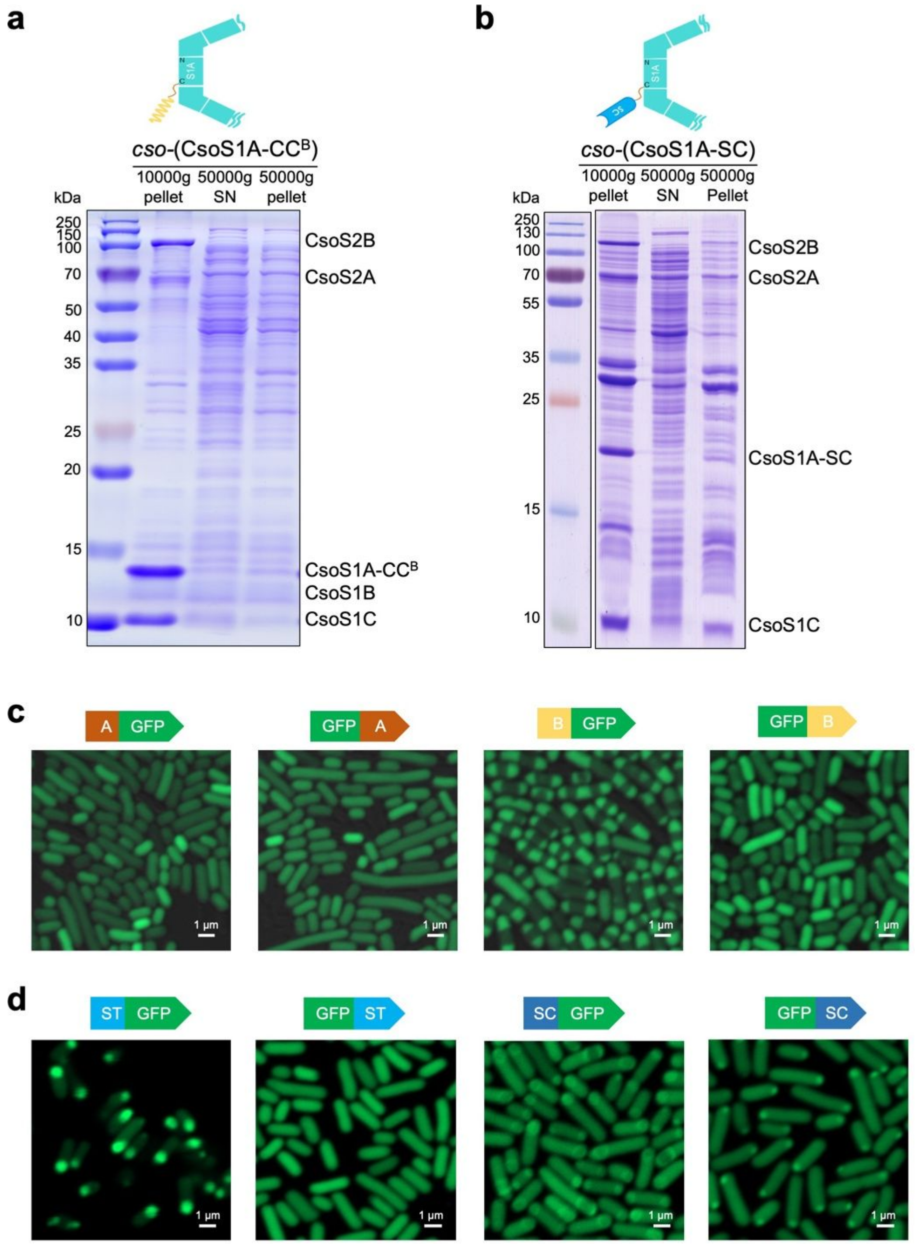

Figure S5. SC or CC^{B} fused at the C-terminus of CsoS1A affect shell formation due to possible self-interaction. SDS-PAGE of the 10,000 × *g* pellet, 50,000 × *g* supernatant and pellet purified from *E. coli* cells expressing *cso*-(S1A-CC^{B}) **(a)** or *cso*-(S1A-SC) operon **(b)**, respectively. **(c)** Confocal images of *E. coli* cells expressing GFP with either CC^{A} or CC^{B} fused to the N- or C-terminus. **(d)** Confocal images of *E. coli* cells expressing GFP with either ST or SC fused to the N- or C-terminus.

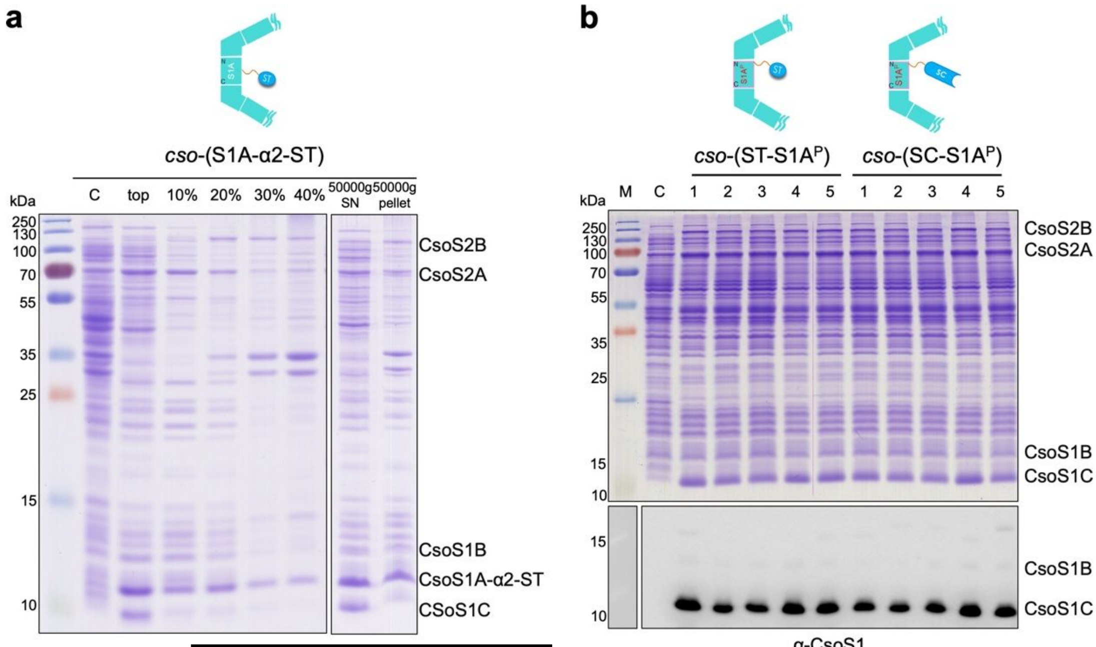

change to "ST/SC insertions"

Figure S6. Other types of insertions on CsoS1A or CsoS1A^P hinder shell formation. (a) SDS-PAGE of 50,000 × *g* supernatant and pellet as well as sucrose fractions purified from cells expressing *cso*-(S1A-α2-ST) operon. **(b)** SDS-PAGE (top) and immunoblot (bottom) of whole cell lysates of cells expressing empty plasmid (control, C) and five independent colonies that expressing *cso*-(ST-S1A^P) and *cso*-(SC-S1A^P), respectively. Immunoblot analysis used anti-CsoS1 antibody.

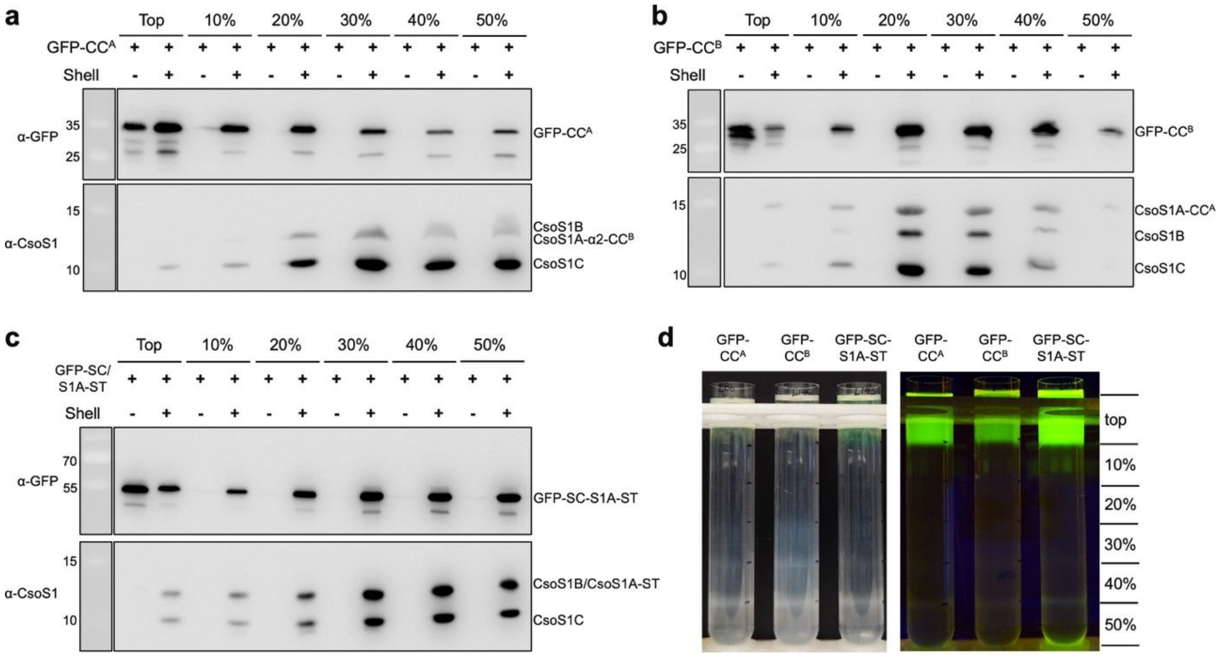

Figure S7. Free GFP-AP was not present in the 10%-50% sucrose fractions. Immunoblot analysis of the top layer and individual sucrose fractions purified from strains producing GFP- CC^A alone or Shell-(GFP-CC^A) assemblies **(a)**, strains producing GFP-CC^B alone or Shell-(GFP-CC^B) assemblies **(b)** and strains producing GFP-SC-S1A-ST or Shell-(GFP-SC) assemblies **(c)**. Immunoblotting was performed using anti-GFP antibody (top) and CsoS1 antibody (below), respectively. **(d)** Sucrose gradient fractions purified from cells expressing GFP-CC^A, GFP-CC^B or GFP-SC-S1A-ST alone under natural light (left) and 480 nm LED illumination (right). Our data show that the GFP-APs alone did not appear in heavier sucrose fractions after sucrose gradient ultracentrifugation, indicating that all GFP-AP constructs did not produce high-density aggregates.

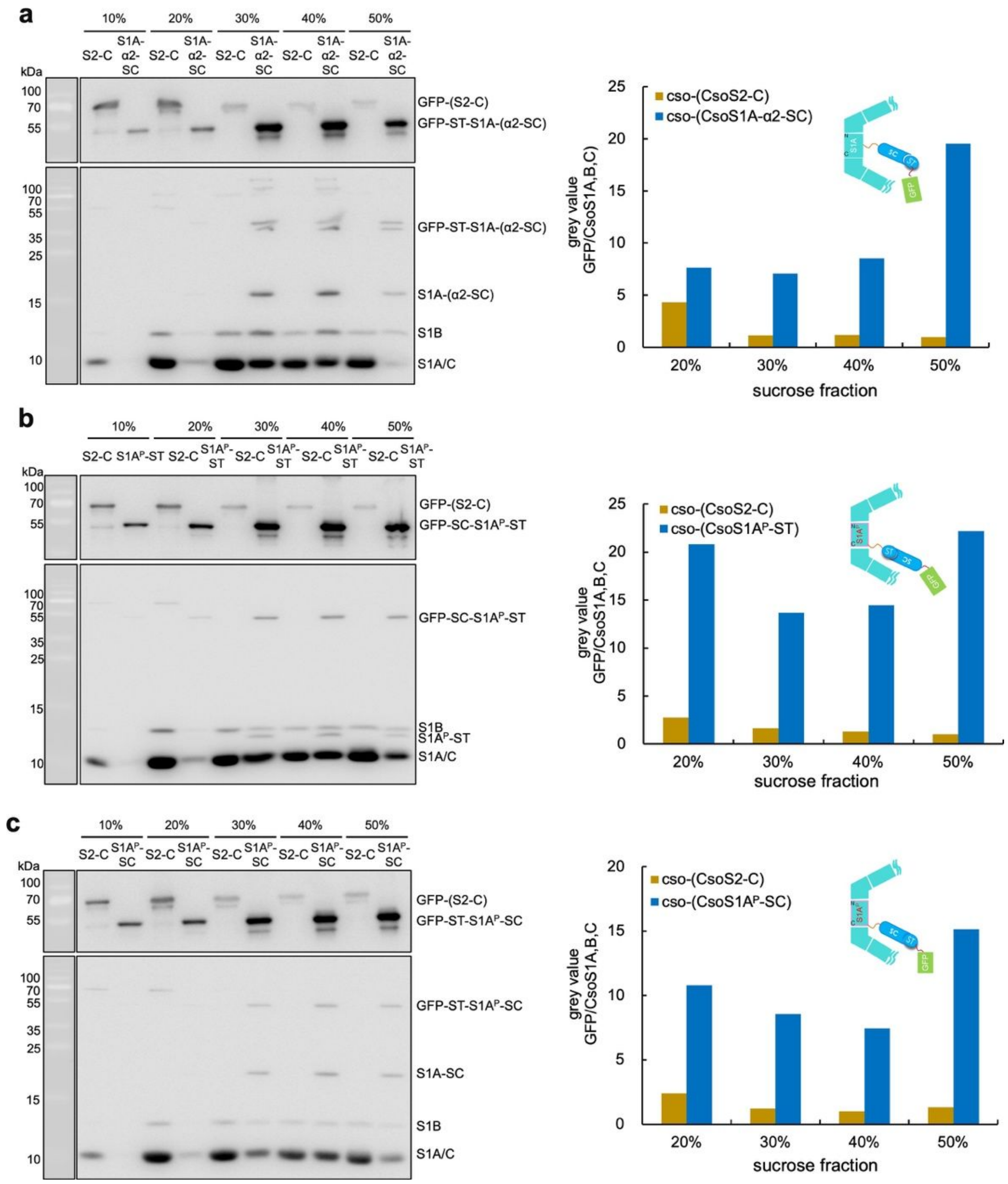

Figure S8. Evaluation of GFP-loading efficiency of the other three types of Spy system-based shells. Immunoblot analysis of samples purified by sucrose gradient centrifugation from *E. coli* cells expressing S1A-α2-SC mediated Shell-GFP assemblies (**a**), S1A^{P}-ST mediated Shell-GFP assemblies (**b**) or S1A^{P}-SC mediated Shell-GFP assemblies (**c**), respectively. Quantification of the ratios of GFP and CsoS1 content in individual sucrose fractions is shown on the right.

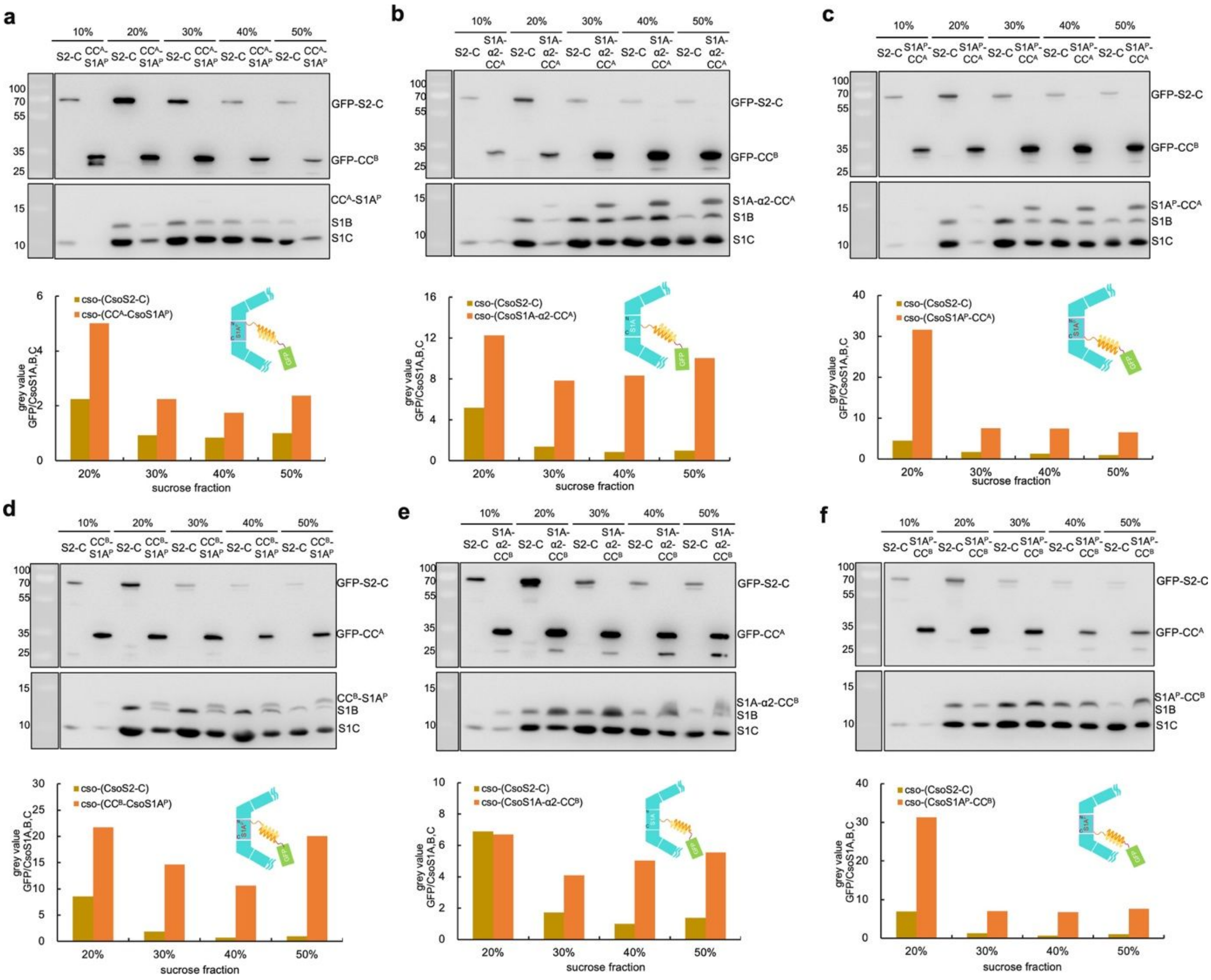

Figure S9. Evaluation of the GFP-loading capacities of the other six types of Coiled-coil system-functionalized shells. Immunoblot analysis of samples purified by sucrose gradient centrifugation from *E. coli* cells expressing CC^A-$S1A^P$-mediated Shell-GFP assemblies (**a**), S1A-α2-CC^A mediated Shell-GFP assemblies (**b**), $S1A^P$-CC^A mediated Shell-GFP assemblies (**c**), CC^A-$S1A^P$ mediated Shell-GFP assemblies (**d**), S1A-α2-CC^B mediated Shell-GFP assemblies (**e**) and $S1A^P$-CC^B mediated Shell-GFP assemblies (**f**), respectively. Quantification of the ratios of GFP and CsoS1 content in individual sucrose fractions is shown at the bottom.

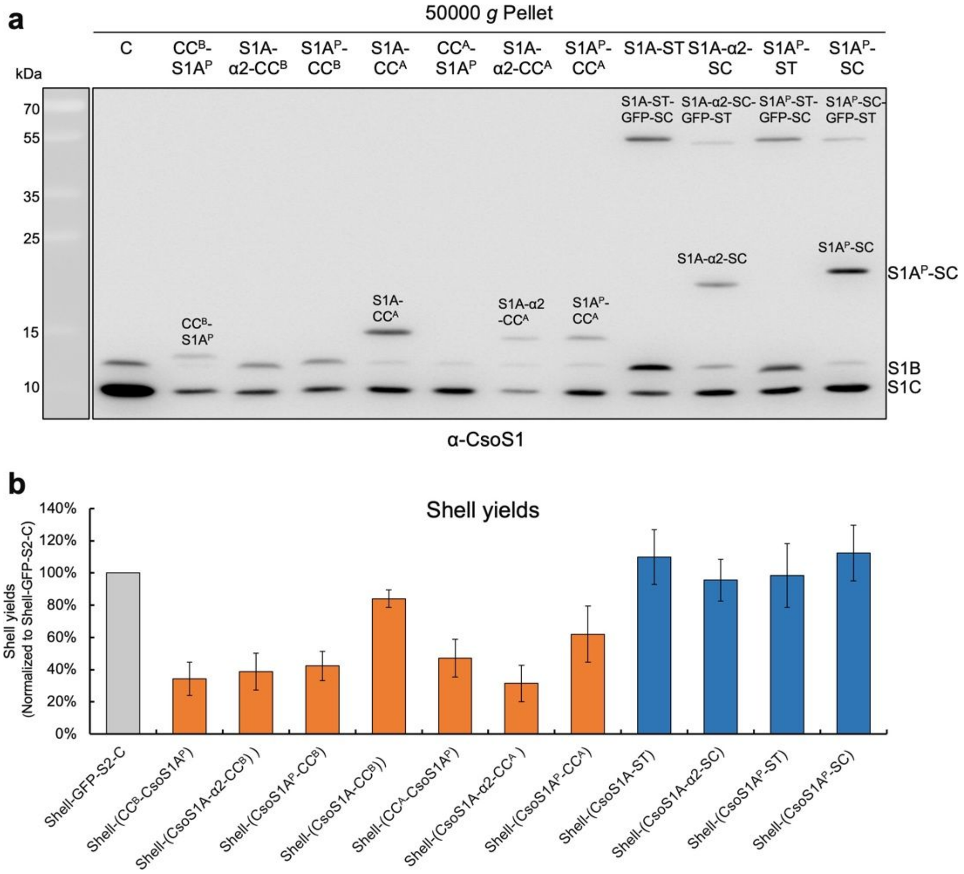

Figure S10. Comparison of the shell yields in cells expressing different types of AP-mediated Shell-GFP assemblies. a. Immunoblot analysis of 50,000 × *g* pellet from *E. coli* cells expressing 11 types of AP-functionalized Shell-GFP assemblies using the anti-CsoS1 antibody. C: WT α-shells as control. **b**. Quantification of the total CsoS1 protein content in individual Shell-GFP assemblies.
